# Alterations in Arbuscular Mycorrhizal Community Along a Chronosequence of Teak (*Tectona grandis*) Plantations in Tropical Forests of China

**DOI:** 10.3389/fmicb.2021.737068

**Published:** 2021-11-26

**Authors:** Zhi Yu, Kunnan Liang, Xianbang Wang, Guihua Huang, Mingping Lin, Zaizhi Zhou, Yinglong Chen

**Affiliations:** ^1^Key Laboratory of State Forestry Administration on Tropical Forestry Research, Research Institute of Tropical Forestry, Chinese Academy of Forestry, Guangzhou, China; ^2^College of Forestry, Nanjing Forestry University, Nanjing, China; ^3^School of Agriculture and Environment, Institute of Agriculture, The University of Western Australia, Perth, WA, Australia

**Keywords:** *Tectona grandis*, arbuscular mycorrhizal fungi, stand age, soil properties, plantations

## Abstract

Arbuscular mycorrhizal (AM) fungi play a crucial role in promoting plant growth, enhancing plant stress resistance, and sustaining a healthy ecosystem. However, little is known about the mycorrhizal status of teak plantations. Here, we evaluated how the AM fungal communities of rhizosphere soils and roots respond to different stand ages of teak: 22, 35, 45, and 55-year-old from the adjacent native grassland (CK). A high-throughput sequencing method was used to compare the differences in soil and root AM fungal community structures. In combination with soil parameters, mechanisms driving the AM fungal community were revealed by redundancy analysis and the Mantel test. Additionally, spore density and colonization rates were analyzed. With increasing stand age, the AM fungal colonization rates and spore density increased linearly. Catalase activity and ammonium nitrogen content also increased, and soil organic carbon, total phosphorous, acid phosphatase activity, available potassium, and available phosphorus first increased and then decreased. Stand age significantly changed the structure of the AM fungal community but had no significant impact on the diversity of the AM fungal community. However, the diversity of the AM fungal community in soils was statistically higher than that in the roots. In total, nine and seven AM fungal genera were detected in the soil and root samples, respectively. The majority of sequences in soils and roots belonged to *Glomus*. Age-induced changes in soil properties could largely explain the alterations in the structure of the AM fungal community along a chronosequence, which included total potassium, carbon-nitrogen ratio, ammonium nitrogen, catalase, and acid phosphatase levels in soils and catalase, acid phosphatase, pH, and total potassium levels in roots. Soil nutrient availability and enzyme activity were the main driving factors regulating the shift in the AM fungal community structure along a chronosequence of the teak plantations.

## Introduction

Tropical forests are one of the most abundant and complex terrestrial ecosystems and play a vital role in maintaining biodiversity and regulating the global climate (Jing et al., 2020). Soil is considered one of the most abundant microbial ecosystems on Earth (Gans et al., 2005). Soil microorganisms, as a crucial component of biodiversity, can promote the decomposition of organic matter, nutrient cycling, and energy flow in terrestrial ecosystems (Chen et al., 2013; Zechmeister-Boltenstern et al., 2015) and are closely related to plant growth. However, relative to the aboveground biomass, the knowledge of the soil microbial community structure and distribution pattern is limited because of its large number and small size (Fitter et al., 2004). Arbuscular mycorrhizal (AM) fungi are widely distributed soil microbes that can form a mutual association with over 80% of terrestrial plants and act as a bridge connecting the belowground to aboveground ecosystems (Wardle et al., 2004; van der Heijden et al., 2015). It is generally accepted that AM fungi can affect plant growth by promoting nutrient uptake, especially phosphorus (P) (Smith and Read, 2008), protecting plants against abiotic stresses, such as drought (Zou et al., 2015; Mo et al., 2016; Ren et al., 2019), salinity (Wu et al., 2015; Chen et al., 2017), heavy metals (Zhang et al., 2020), low temperature (Zhou et al., 2012b), protection of host plants from pathogenic infections (Lin et al., 2021), improving soil aggregate stability by secreting glomalin (Zou et al., 2014; Ji et al., 2019), and affecting ecosystem productivity (van der Heijden et al., 2008; Zhu et al., 2017). However, these physiological and ecological functions of AM fungi are highly dependent on species diversity and community composition (Jiang et al., 2018). A case in point was reported by van der Heijden et al. (1998), who found that in the grassland ecosystem, inoculation of 14 AM fungal species increased plant diversity by 105% and plant productivity by 42% compared to that in the ecosystem inoculated with only one AM fungal species. A study has also revealed that different AM fungi can absorb P at different distances from the root of the plant, making the host plants inoculated with mixed AM fungal species grow better than those inoculated with AM fungal species (Smith et al., 2000). Thus, some scholars put forward the theory of “functional complementarity” to explain the above phenomena. With an increase in the richness of AM fungi, the complementary functions of AM fungi promote plant growth better (Smith et al., 2000). Therefore, a growing number of studies are focused on the diversity and mechanisms of the assembly of AM fungal communities in ecosystems and the exploration of factors shaping AM fungal community composition.

Generally, the factors driving the diversity and structure of AM fungal communities can be divided into three categories: abiotic and biotic environmental factors, and random factors (Vályi et al., 2016). Previous studies have reported that the structural shifts in AM fungal communities were related to the carbon-nitrogen ratio (C/N) and pH in an experiment of nitrogen and phosphorus addition (Wang et al., 2020); C/N ratio and light intensity in an experiment of shading treatment (Shi et al., 2014); soil moisture, fine root biomass, and pH in an experiment of warming treatment in a young subtropical *Cunninghamia lanceolata* plantation (Cao et al., 2020); and soil moisture, soil organic carbon, and total nitrogen along a large-scale aridity gradient (Yang et al., 2011). The succession of taxa in a community over time is a fundamental attribute of the ecosystem (Chen et al., 2000; Gossner et al., 2016). Afforestation is an effective restoration technique to restore degraded forest ecosystems by increasing vegetation and biodiversity (Wang et al., 2021). It can change the composition and diversity of vegetation flora, soil physicochemical parameters, microclimate in the forest, and accumulation and decomposition of litter through ecological succession (Zhao et al., 2018; Xu M.P. et al., 2020). Thus, the shifts mentioned above may have influenced the AM fungal community. Many studies have demonstrated that the shift in the AM fungal community over ecological succession is associated with variations in soil properties induced by stand age, such as soil texture (Herrmann et al., 2016), pH (Reyes et al., 2019), and nutrient availability (Wang et al., 2020). Usually, primary productivity is initially limited by nitrogen (N) in young soils, which enters the ecosystem mainly *via* biological nitrogen fixation, but productivity reaches the highest level relatively quickly. In contrast, P has become increasingly limited in old soils as the amount and availability of P declines over time (Krüger et al., 2015). The recipient symbiosis between plants and AM fungi is based on the carbon cycle, specifically fatty acids (Jiang et al., 2017). It is well established that plant communities become more complex with succession. Existing studies have pointed out that up to 30% of the carbon produced by photosynthesis of plants is allocated to the symbiotic AM fungal community, which is stored in the hyphae or secreted into the soil (Drigo et al., 2010). Meanwhile, the quality and quantity of litter and root exudates will alter with plant succession, resulting in the accumulation of soil organic carbon and driving the improvement of soil physicochemical properties (Zhu et al., 2012). Therefore, AM fungal communities may be influenced by variations in plant community composition, growth, and development. For example, the developmental stages of host plant and plant communities also affect the composition and diversity of AM fungi (Chen et al., 2000; Hart et al., 2014). Furthermore, some researchers found that the composition variation increased along the chronosequence, but the richness of AM fungal taxa was unaffected by host plant age (Krüger et al., 2017). This phenomenon may be attributed to the dispersal speed of AM fungi, which seem to lack dispersal limitation on larger spatial and temporal scales (Davison et al., 2015). Thus, AM fungal dispersal and establishment at different sampling sites were performed before the investigation. The variation in AM fungal composition was significantly correlated with the composition of neighboring plant species rather than soil chemical parameters, suggesting that AM fungal communities seemed to be affected by biotic rather than abiotic factors (Krüger et al., 2017).

Teak (*Tectona grandis* L.f.) is widely cultivated worldwide due to its excellent timber quality, high market demand, and good economic and social value (Zhou et al., 2017; Huang et al., 2019). As a tropical hardwood tree species, teak is naturally distributed in India, Myanmar, Laos, and Thailand (Yang et al., 2020). Teak was introduced into tropical China 170 years ago, and 60% of the soils in these planted areas are acidic or severely acidic (pH < 5.5), resulting in a deficiency of available nutrients affecting its growth (Zhou et al., 2012a). Studies have shown that teak can be inoculated with AM fungi (Zhou et al., 2012b; Chaiyasen et al., 2014, 2017). Inoculation with AM fungi significantly increases the transplanting survival rate and promotes the growth and quality of teak seedlings (Chaiyasen et al., 2017). For instance, the inoculation of *Glomus aggregatum* accelerated the height and diameter growth of teak seedlings by 61% (Irianto and Santoso, 2005). Moreover, a study found that the symbiotic efficiency of different AM fungal species toward teak varied greatly in terms of growth parameters and nutritional status (Rajan et al., 2000). Consequently, it is meaningful to study the shift in the AM fungal community with succession for screening efficient AM fungi for teak. However, little information is available on the diversity of AM fungal communities and changes in the community structure over a period of time after afforestation. Considering the vital physiological and ecological functions of AM fungi in promoting plant growth and nutrient absorption, it is necessary to investigate AM fungal association with teak, screen the potential AM fungal isolates, and provide insights for the development of biological fertilizer for teak.

Using high-throughput sequencing, this study investigated AM fungal communities in four teak plantations of varying ages and the adjacent native grassland in Mt. Jianfengling, Hainan Province, China. The objectives of this study were (i) to analyze the AM fungal community characteristics and dynamics with increasing stand ages of teak plantations, and (ii) to explore key edaphic factors driving variation in the AM fungal community.

## Materials and Methods

### Study Location and Climate

The study was conducted at Mt. Jianfengling (18°20′–18°57′ N, 108°41′–109°12′ E) in Hainan, China. This region is situated at an altitude of 60–750 m and is characterized by a tropical monsoon climate with an annual mean temperature of 24.5°C. The annual maximum temperature is 38.1°C and the minimum temperature is 2.5°C. The average annual rainfall is 1649.9 mm with 80–90% of rainfall occurring from May to October, especially in August and September. The relative humidity varies between 80 and 88%, and the soil type is yellow latosol.

### Experimental Design and Sampling

Teak plantation chronosequence included four age groups of 22, 35, 45, and 55 years, which were planted in 1998, 1985, 1975, and 1965, respectively. In addition, nearby native grassland with no history of the teak plantation was sampled as a control and marked as CK (Supplementary Figure 1 and Table 1). Three standard sites with dimensions of 20 m × 20 m were set up for each age group. The distance between these standard sites was less than 100 m, and the stand distance of different ages was less than 5 km (Table 1). Sampling was conducted in 2020, and rhizosphere soil and fine roots were collected. Five dominant teak trees in each standard site were selected as the research objects. The top soil at a depth of 5 cm was removed from each selected site to expose the roots. Each root was followed to its origin, and the fine roots (<2 mm diameter) were collected using a shovel by gentle shaking to remove the soil (5–15 cm) tightly attached to it. The rhizosphere soil was obtained by gently brushing the root surface with a sterile brush. All samples of fine roots or rhizosphere soil from each site were mixed to form a composite sample for the roots or soil. The rhizosphere soil was divided into two parts, one part was used for soil property analysis and AM fungal spore density determination by passing through a 2 mm sieve after air drying, and the other part and fine roots were stored in a deep freezer at −80°C for DNA extraction. Finally, 15 soil samples (12 from teak plantations of four age groups and three from the CK) and 12 root samples (no teak roots in the CK) were obtained.

**TABLE 1:** Characteristics of different stands of teak plantations.

Sites	Mean DBH (cm)	Mean height (m)	Canopy closure (%)	Altitude (m)	Slope degree (°)	Dominant species in the understory
CK	–	–	–	118	11	*H. contortus*
22 Y	16.9	21.1	0.70	142	10	*E. odoratum*, *B. cristata*, *C. microphylla*
35 Y	20.3	32.9	0.76	104	17	*A. dioica*, *L. leucocephala*, *T. planicaule*
45 Y	23.4	35.5	0.67	96	13	*L. leucocephala*, *A. pavonlna*
55 Y	24.5	39.7	0.73	135	8	*E. odoratum*, *L. coromandelica*, *G. lobbianum*, *C. microphylla*

*Values represent means (n = 3). DBH, diameter at breast height; H. contortus, Heteropogon contortus; E. odoratum, Eupatorium odoratum; B. cristata, Barleria cristata; C. microphylla, Carmona microphylla; A. dioica, Aporusa dioica; L. leucocephala, Leucaena leucocephala, T. planicaule, Tetrastigma planicaule; A. pavonlna, Adenanthera pavonlna; L. coromandelica, Lannea coromandelica; and G. lobbianum, Gonocaryum lobbianum. 22Y, 22-year-old stand; 35Y, 35-year-old stand; 45Y, 45-year-old stand; 55Y, 55-year-old stand; CK, control.*

### Soil Properties and Analysis

The pH of the soil suspension (water ratio of 1: 2.5, w/v) was assessed using a glass electrode pH meter. The total organic carbon (TOC) content was determined using the high-temperature external heat dichromate oxidation capacity method (Schinner et al., 1996). Total nitrogen (N) was measured using the Kjeldahl method. The soil total phosphorous (P), total potassium (K), and available potassium (AK) content were measured using the methods reported by Ma et al. (2020). Soil available phosphorous (AP) was extracted by using HCl-NH_4_F solution and determined by the molybdenum-antimony resistance colorimetric method. The soil ammonium-N and nitrate-N were extracted with the KCl solution and measured using indophenol blue colorimetry and two-wavelength ultraviolet spectrometry method (Liu et al., 2020). The urease, acid phosphatase, and catalase activities were measured as described by Zhen et al. (2019).

### Arbuscular Mycorrhizal Fungal Colonization and Spore Density

Subsamples of 5.0 g fine roots were stained with 0.05% trypan blue using the methods of Phillips and Hayman (1970), and the AM fungal colonization rate was determined under a stereomicroscope at 10 × 20 magnification according to the gridline intersection method described by Giovannetti and Mosse (1980). AM fungal spore density was measured as described by Sheng et al. (2017). AM fungal spores were extracted by wet sieving and decanting with 10 g of frozen rhizosphere soil, and then density gradient centrifugation was performed using a 50% sucrose solution. The spores were placed on a white gridded cellulose nitrate filter (1.2-um pore size) with a pipette, washed with distilled water, and spread evenly. The spores that appeared to be viable (based on color, shape, surface condition, and spore contents) were counted under a compound microscope (Olympus DP70, Japan) at 10 × 10 magnification.

### DNA Extraction and Polymerase Chain Reaction Amplification

The DNA in the soil and root samples was extracted using the E.Z.N.A.^®^ soil DNA Kit (Omega Bio-tek, Norcross, GA, United States) according to the manufacturer’s protocols. The concentration and purification of the extracted products were determined using a NanoDrop 2000 UV-Vis spectrophotometer (Thermo Fisher Scientific, Wilmington, United States). DNA quality was checked using a 1% agarose gel. The partial small subunit region of the 18S rRNA gene was amplified by nested polymerase chain reaction (PCR) using AML1/AML2 (5′-ATCAACTTTCGATGGTAGGATAGA-3′; 5′-GAACCCAAACACTTTGGTTTCC-3′) as the first primer set using a thermocycler PCR system (GeneAmp 9700, ABI, United States) (Lumini et al., 2010). The amplification reaction mixture contained: 5 × *TransStart* FastPfu buffer (4 μL), 2.5 mM dNTPs (2 μL), 5 μM forward primer (0.8 μL), 5 μM reverse primer (0.8 μL), *TransStart* FastPfu DNA polymerase (0.4 μL), bovine serum albumin (0.2 μL), and template DNA (10 ng), and ddH_2_O was added to make a final volume of 20 μL. The PCR amplification conditions were as follows: initial denaturation at 95°C for 3 min, followed by 32 cycles of denaturation at 95°C for 30 s, annealing at 55°C for 30 s, and extension at 72°C for 45 s, and a final extension at 72°C for 10 min. AMV4.5NF/AMDGR (5′-AAGCTCGTAGTTGAATTTCG-3′; 5′-CCCAACTATCCCTATTAATCAT-3′) was used as the second primer set in the second round of PCR using the same procedure described above (Ji et al., 2020), except for the number of amplification cycles, which was 30. Each sample was repeated three times.

### Illumina Miseq Sequencing

According to the manufacturer’s protocols, 2% agarose gel was used to extract the PCR amplification product, which was purified using an AxyPrep DNA gel extraction kit (Axygen Biosciences, Union City, CA, United States). The products were quantified using a Quantus Fluorometer (Promega, United States). Thereafter, purified amplicons were pooled in equimolar amounts and paired-end sequenced on an Illumina MiSeq PE300 platform (Illumina, San Diego, United States). The raw reads for all samples were sent to the NCBI Sequence Read Archive (SRA) database (accession number: PRJNA743782).

### Analysis of Sequencing Data

Trimmomatic was used to demultiplex the raw fastq files and for quality filtering, and the reads were merged using FLASH version 1.2.7. The chimeric sequences were identified and removed, and the cleaned sequences were clustered into operational taxonomic units (OTUs) based on 97% similarity using UPARSE (version 7.1).^1^ The most abundant sequence in an OTU was considered a representative unit. The taxonomy of each OTU representative sequence was analyzed by RDP Classifier (version 2.2)^2^ against the Sliva database (release 138)^3^ and the MaarjAM database^4^ using a confidence threshold of 0.9.

### Statistical Analysis

One-way analysis of variance and multiple significant differences (DMRT; *P* < 0.05) were used to compare the changes in soil properties, diversity indices, and relative abundance. The diversity indices and relative abundance were normalized with log _10_ (X + 1) conversion before assessment. Regression analysis was used to test the relationships among stand age, AM fungal spore density, and colonization levels using Origin 2021 (Origin Lab Corp., Northampton, MA, United States). Sobs (observed richness), Chao 1 (Chao 1 estimator), Shannon (Shannon diversity index), and coverage (Good’s coverage indices) were calculated using Mothur (version v.1.30.1),^5^ which were used to assess sample richness, diversity, and coverage. Venn and UpSet diagrams were drawn using the R package Vegan and UpSet. The community composition analysis was conducted and figures were generated using the R-package Vegan. The differences in the composition of AM fungal communities were analyzed based on the non-metric multidimensional scaling (NMDS) of Bray-Curtis distances. In addition, the analysis of similarities (ANOSIM) and the permutation multivariate analysis of variance (PERMANOVA) were conducted to test the statistically significant differences among AM fungal communities of different stand ages. The relationships among diversity, richness, spore density, colonization rate, and soil properties were analyzed using Pearson’s correlation. The relationship between the composition and soil parameters was analyzed using Spearman correlation, and a heatmap was drawn using the R package pheatmap. The Mantel test was applied to assess the effects of soil on AM fungal community composition at the OTU level. Redundancy analysis was used to study the soil factors that have a significant effect on the AM fungal community using Canoco 5.0 software (Microcomputer Power, Inc., Ithaca, NY, United States).

## Results

### Soil Characteristics

Soil chemical properties changed significantly along the chronosequence of teak plantations (*P* < 0.05; Table 2). Soil parameters, such as TOC, P, AP, AK, C/N ratio, and urease activity, first increased and then decreased. The TOC, P, AP, and urease activity peaked at 35Y, and the AK and C/N ratios peaked at 45Y. The C/P ratio and NO_3_^–^-H did not show significant difference with the increase in stand age. The rhizosphere soil was slightly acidic, with a pH ranging from 6.53 to 6.77. Compared to those in the CK, N and NH4^+^-H contents increased as stand age progressed, whereas K content decreased. Catalase and soil acid phosphatase activities were also significantly affected by stand age (*P* < 0.05), which was higher than that of the CK.

**TABLE 2:** Soil properties in the rhizosphere of 22, 35, 45, and 55-year-old teak plantations in an adjacent grassland (CK) in Mt. Jianfengling, Hainan island, China.

Soil properties	CK	22-year-old	35-year-old	45-year-old	55-year-old
Ph	6.53 ± 0.038^b^	6.40 ± 0.052^c^	6.56 ± 0.012^b^	6.56 ± 0.009^b^	6.77 ± 0.007^a^
TOC (g/kg)	26.07 ± 1.123^b^	28.59 ± 1.774^b^	54.44 ± 5.082^a^	44.85 ± 3.951^a^	32.43 ± 1.213^b^
N (g/kg)	1.45 ± 0.039^c^	1.69 ± 0.059^c^	3.03 ± 0.189^ab^	1.81 ± 0.071^bc^	3.15 ± 0.869^a^
P (g/kg)	0.28 ± 0.005^b^	0.25 ± 0.023^b^	0.43 ± 0.056^a^	0.32 ± 0.006^b^	0.31 ± 0.012^b^
C/P	92.03 ± 2.375^a^	117.14 ± 9.793^a^	133.34 ± 30.17^a^	140.11 ± 11.66^a^	104.76 ± 4.002^a^
C/N	18.01 ± 0.485^b^	16.87 ± 0.59^b^	17.9 ± 0.76^b^	24.75 ± 1.775^a^	11.57 ± 2.377^c^
K (g/kg)	38.6 ± 0.174^a^	21.38 ± 0.327^d^	19.98 ± 0.701^d^	34.85 ± 1.413^b^	31.94 ± 0.928^c^
AP (mg/kg)	2.35 ± 0.116^ab^	1.42 ± 0.094^b^	3.31 ± 0.503^a^	1.68 ± 0.345^b^	1.42 ± 0.396^b^
AK (mg/kg)	78.8 ± 7.833^c^	135.69 ± 19.132^c^	146.77 ± 5.332^c^	411.35 ± 84.651^a^	287.15 ± 7.666^b^
NO_3_^–^H (mg/kg)	13.98 ± 1.582^a^	15.02 ± 0.517^a^	14.67 ± 1.049^a^	18.47 ± 2.696^a^	17.26 ± 1.247^a^
NH_4_^+^-H (mg/kg)	9.15 ± 0.623^c^	10.87 ± 0.297^bc^	11.22 ± 0.455^bc^	15.19 ± 2.505^ab^	19.16 ± 2.69^a^
Catalase (mL/g)	9.78 ± 1.576^d^	14.68 ± 0.341^bc^	14.21 ± 0.655^c^	17.8 ± 1.328^b^	23.34 ± 0.777^a^
Acid phosphatase (mg/g)	0.43 ± 0.023^d^	0.51 ± 0.013^d^	1.09 ± 0.027^a^	0.9 ± 0.049^b^	0.67 ± 0.014^c^
Urease (mg/g)	5.78 ± 0.296^b^	1.52 ± 0.012^c^	10.2 ± 0.7^a^	2.14 ± 0.249^c^	1.13 ± 0.159^c^

*Values are expressed as mean ± standard error (n = 3) in each line followed by different letters indicating a significant difference (P < 0.05); C/P = TOC/P, C/N = TOC/N; NH_4_^+^-H, ammonium nitrogen; NO_3_^–^-H, nitrate nitrogen; TOC, total organic carbon; N, soil total nitrogen; P, soil total phosphorus; K, soil total potassium; AP, available phosphorus; AK, available potassium.*

### Arbuscular Mycorrhizal Fungal Colonization and Spore Density

**FIGURE 1:**
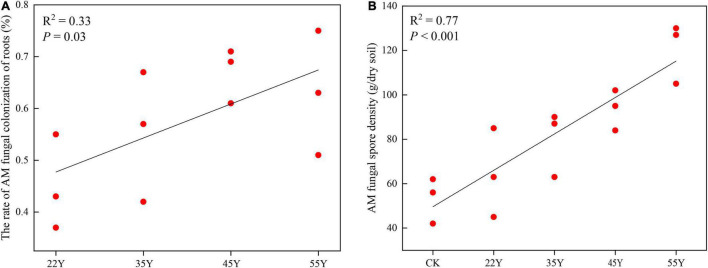
Relationship between teak stand ages and AM fungal colonization rates in teak roots **(A)**, AM fungal spore density **(B)**. 22Y, 22-year stand; 35Y, 35-year stand; 45Y, 45-year stand; 55Y, 55-year stand; CK: control.

Mycorrhizal colonization of teak roots was observed in all the treatments. Mycorrhizal colonization rates and spore density increased linearly among chronosequences (Figures 1A,B). The range of colonization in roots and spore density in soils varied from 0.45 ± 0.03 to 0.63 ± 0.04 and from 53.33 ± 3.42 to 120.67 ± 4.55, respectively. The Pearson correlation analysis showed that spore density was positively correlated with pH, N, AK, NH_4_^+^-H, and catalase, whereas colonization was negatively correlated with K, NO_3_^–^-H, NH_4_^+^-H, and catalase (Supplementary Table 1).

### Sequence Summary and Arbuscular Mycorrhizal Fungal Diversity Indices in Soil and Root

**FIGURE 2:**
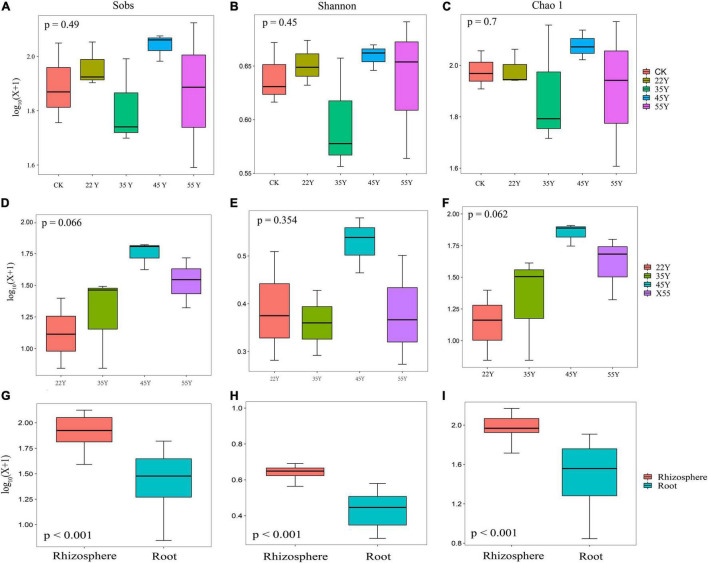
AM fungal community diversity in rhizosphere soils **(A–C)** and roots **(D–F)** along stand age (*n* = 3). Alpha diversity indices **(G–I)** of AM fungal communities were compared between rhizosphere (*n* = 15) and root (*n* = 12) samples. All the diversity indices (at 97% sequence similarity) are delineated at the OUT level.

A total of 351,843 (range: 20,492–24,792) and 250,065 (range: 20,301–23,884) high-quality 18S rDNA sequences belonging to *Glomeromycota* were amplified from 15 soil and 12 root samples, respectively (Supplementary Table 2). Based on the 97% sequence similarity, the sequences obtained in soil and root samples were clustered into 367 and 217 AM fungal OTUs, and 195 OTUs were shared between the soil and root. The unique OTUs detected in soil samples (172) far exceeded those in the root samples (22) (Supplementary Figure 2). The results from the rarefaction curves showed that the curve gradually flattened as the number of sampling reads increased, which indicated that the sequencing depth was sufficient to reflect the AM fungal diversity, and it also highlighted higher AM fungal diversity in soil than in the roots (Supplementary Figure 3). We found no significant differences in the diversity (Shannon) and richness (Sobs and Chao 1) of the AM fungal communities in the rhizosphere soil and roots with increasing stand age (Figure 2), indicating that stand age had no significant effect on the diversity and richness of the AM fungal community. However, the diversity and richness of the rhizosphere soils were significantly higher than those of the roots (*p* < 0.001; Figure 2). Coverage was above 99.9% for each sample, indicating that AM fungal communities were well sampled owing to the more depth of Illumina sequencing (Supplementary Table 2).

### Arbuscular Mycorrhizal Fungal Community Composition and Structure in Rhizosphere Soil and Root

**FIGURE 3:**
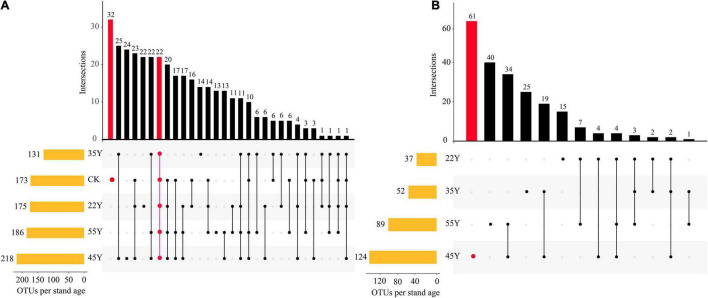
UpSet plots show the relationships of AM fungi OTUs between rhizosphere soils **(A)** and roots **(B)** of teak along a chronosequence. The left panel displays orange bars for each stand age that represent the number of AM fungi OTUs. Dark circles in the matrix indicate sets of treatment that intersect. The two intersection queries are the one-way intersection of CK (red) and the five-way intersection of all 5 stand ages in the rhizosphere soils. The one intersection query is the one-way intersection of 45Y (red) in the roots.

The distribution of OTUs among the different stand ages is shown in the UpSet diagram (Figure 3). In the rhizosphere soil samples, the results showed that the number of AM fungal OTUs in 45Y was the highest (218; 59.4% of all 367 OTUs), followed by 55Y (186; 50.7%), 22Y (175; 47.7%), CK (173; 47.1%), and 35Y (131; 35.7%). In addition, the number of AM fungi OTUs unique to CK was the highest (32; 8.7%), followed by 45Y (24; 6.5%), 22Y (22; 6.0%), 35Y (14; 3.8%), 55Y (0). 22 OTUs (6.0%) were common to all five soil periods of chronosequence (Figure 3A). In the root samples, the results showed that the number of AM fungal OTUs in the 45Y was the highest (124; 57.1%), followed by 55Y (89; 41.0%), 35Y (52; 24.0%), and 22Y (37; 17.1). The number of AM fungi OTUs unique to 45Y was also the highest (61; 28.1%).

**FIGURE 4:**
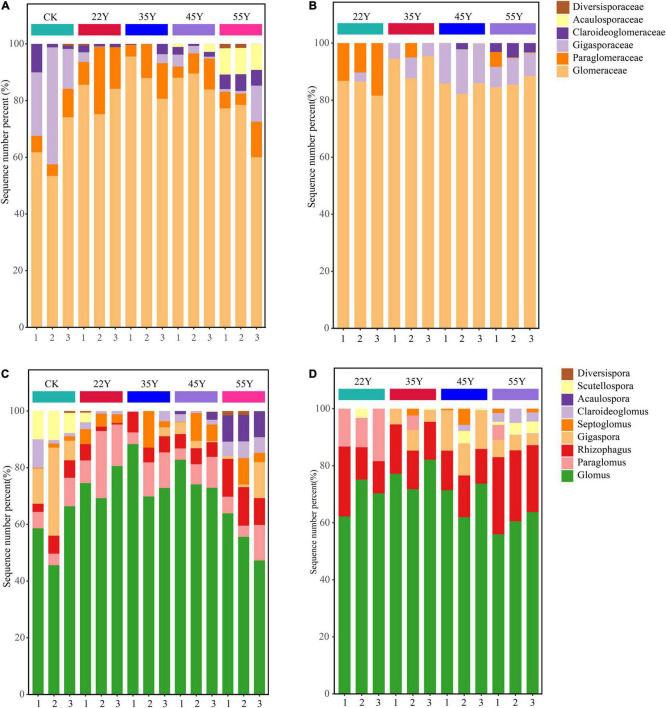
The relative abundance of AM fungal community composition at the family **(A,B)** and genera **(C,D)** levels in the rhizosphere soils **(A,C)** and in the roots **(B,D)** along a teak plantation chronosequence. Values are mean ± standard error (*n* = 3).

**FIGURE 5:**
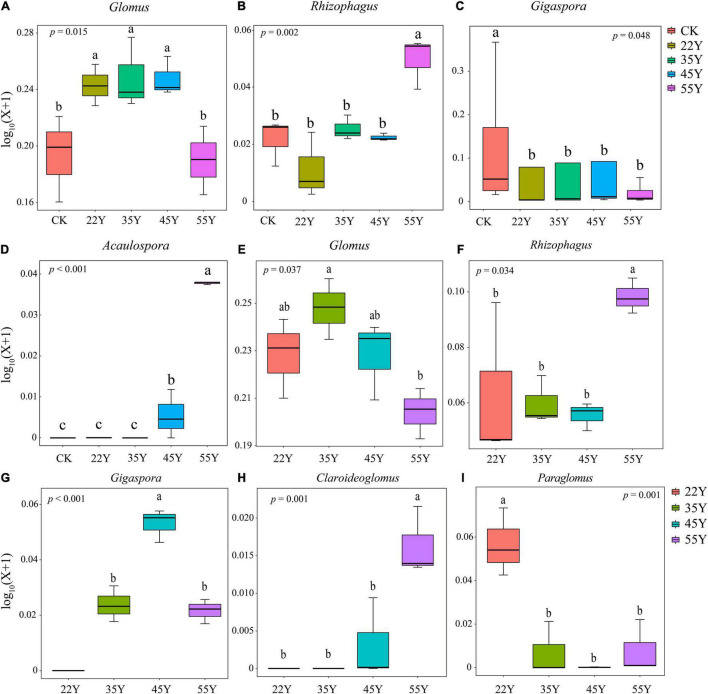
The relative abundance of AM fungal genus in the root **(A–D)** and the rhizosphere soil **(E–I)**. Lowercase letters indicate significant differences among different stand ages (*P* < 0.05). 22Y, 22-year stand; 35Y, 35-year stand; 45Y, 45-year stand; 55Y, 55-year stand; CK, control.

As shown in Figure 4, AM fungi were assigned to a total of six families in the rhizosphere soil, including *Glomeraceae*, *Paraglomeraceae*, *Gigasporaceae*, *Claroideoglomeraceae*, *Acaulosporaceae*, and *Diversisporaceae*. *Glomeraceae* (78.39%) was the most abundant family in all stand ages, followed by *Paraglomeraceae* (9.29%) (Figure 4A). Similarly, AM fungi were assigned to four families in the root, including *Glomeraceae* (87.11%), *Gigasporaceae* (7.43%), *Paraglomeraceae* (4.39%), *and Claroideoglomeraceae* (1.13%) (Figure 4B). At a higher resolution, *Glomus* was the dominant AM fungal genus in the rhizosphere soil and root, accounting for 68.19 and 68.89%, respectively. However, the sub-dominant genera between rhizosphere soils and roots were different, that is, *Paraglomus* (9.29%) in the rhizosphere soil samples and *Rhizophagus* (17.28%) in root samples (Figures 4C,D and Supplementary Table 4). The stand age had a significant effect on the composition of the AM fungal community (Figure 5). The relative abundance of *Glomus* first increased and then decreased, peaking at 35Y. The relative abundance of *Rhizophagus* and *Acaulospora* increased with stand age, whereas the relative abundance of *Gigaspora* showed the opposite tendency. In the roots, the relative abundance of *Glomus* and *Gigaspora* initially increased and then declined, peaking at 35Y and 45Y, respectively. The relative abundance of *Rhizophagus* and *Claroideoglomus* increased with succession. However, the relative abundance of *Paraglomus* decreased significantly with increasing stand age.

**FIGURE 6:**
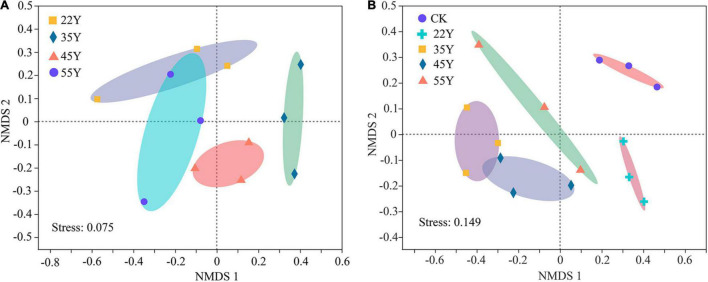
Non-metric multidimensional scaling (NMDS) ordinations of the AM fungal community compositions (Bray-Curtis) in the roots **(A)** and the rhizosphere soil **(B)** based on the abundance of OTUs among different stand ages. 22Y, 22-year stand; 35Y, 35-year stand; 45Y, 45-year stand; 55Y, 55-year stand; CK, control.

The NMDS ordination showed that soil sample sites were differentiated into four clusters, which showed clear separation of community composition among different stand ages except 45Y and 35Y sites. The root sample sites clustered into three groups by the NMDS ordination, except for the 22Y and 55Y sites partly overlapping (Figure 6). Analysis of ANOSIM and PERMANOVA demonstrated that AM fungal community structure significantly varied among samples from different stand ages in soil (*P* = 0.001; *P* = 0.001) and roots (*P* = 0.022; *P* = 0.025) (Table 3), which supported the results of NMDS ordination analysis.

**TABLE 3:** Mantel analysis of the relationship between relative abundance of OTUs and soil parameters.

Variables	Rhizosphere soil		Root	
	*R* ^2^	*P*	*R* ^2^	*P*
Ph	0.164	0.095	0.241	0.068
TOC	0.388	**0.002**	0.359	**0.009**
N	0.251	**0.015**	0.304	0.050
P	0.187	0.065	0.212	0.130
C/P	0.072	0.255	0.335	**0.037**
C/N	−0.188	0.955	0.100	0.298
K	0.130	0.086	0.053	0.315
AP	0.140	0.113	0.194	0.113
AK	0.030	0.366	0.026	0.405
NO_3_^–^H	−0.154	0.921	0.057	0.370
NH_4_^+^-H	0.028	0.400	0.074	0.334
Catalase	0.180	0.051	0.015	0.428
Acid phosphatase	0.587	**0.001**	0.485	**0.001**
Urease	0.292	**0.011**	0.303	**0.043**

*P-values in bold are statistically significant (p < 0.05).*

### Relationships Between Soil Characteristics and Arbuscular Mycorrhizal Fungal Community

**FIGURE 7:**
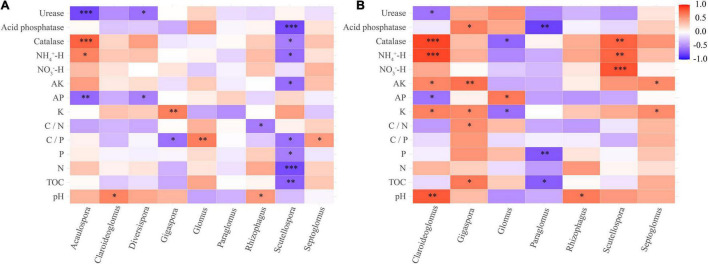
Correlation heat map between soil chemical properties and AM fungal community (genus level) of rhizosphere soil **(A)** and root **(B)** of the teak plantations with different stand ages. *p < 0.05; ^**^p < 0.01; ^***^p < 0.001.

**FIGURE 8:**
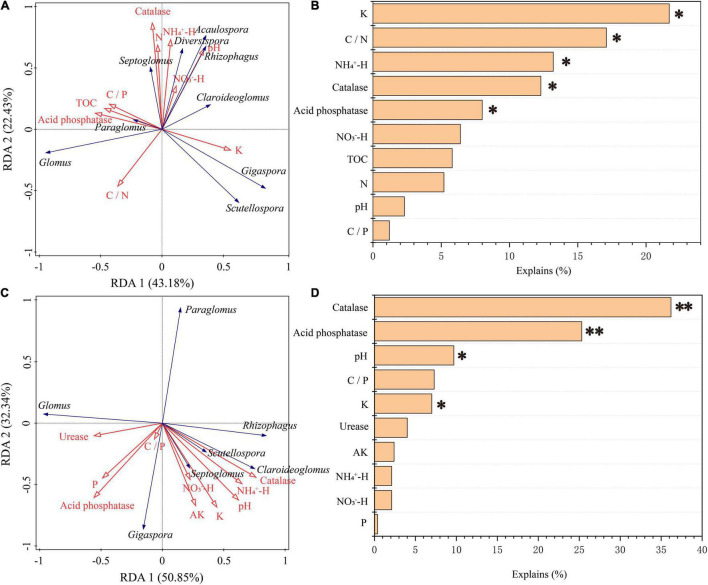
Ordination plots of the redundancy analysis (RDA) showing the relationships between soil chemical properties (red arrows) and the AM fungal genera (blue arrows) in roots **(C)** and in the rhizosphere soils **(A)**. The contributions of soil chemical properties to the variations for AM fungal communities in the rhizosphere soil **(B)** and root **(D)**. *p < 0.05, ^**^p < 0.01.

The correlation heat map showed that the abundance of AM fungi in the rhizosphere soil was not significantly correlated with the NO_3_^–^-H content (*P* > 0.05). The C/P ratio and N content were not statistically correlated with the abundance of AM fungi in the roots (Figure 7). Pearson’s correlation test showed that the Sobs and Chao 1 indices of root-associated AM fungal communities were significantly positively related to urease activity and AP content. The P content was statistically positively related to the Shannon index. The Sobs and Chao 1 indices were significantly negatively correlated with K content and catalase activity (Supplementary Table 3). Redundancy analysis and Monte Carlo permutation test showed that K, NH_4_^+^-H content, C/N ratio, catalase, and acid phosphatase activities were factors affecting the soil AM fungal community structure (Figures 8A,B). The pH, K content, catalase, and acid phosphatase activities were the main factors affecting the structure of the root-associated AM fungal community (Figures 8C,D). The Mantel test showed that the structure of the soil AM fungal community was significantly correlated with TOC, N content, acid phosphatase, and catalase activities. The structure of the root-associated AM fungal community was statistically related to the TOC content, C/P ratio, acid phosphatase, and urease activities (Table 3).

## Discussion

### Effects of Stand Age on Rhizosphere Soil Properties of Teak Plantations

Soil properties changed along a chronosequence of the teak plantations, especially TOC, N, P, AK, and AP (Table 2). Afforestation caused an increase in the above nutrients, followed by a decline. The vegetation and biomass in the teak plantation gradually increased, and the nutrients in the soil accumulated through decomposition of the litter and root exudates (Zhang et al., 2016). This change may lead to an increase in soil nutrients in the later stage of succession. The decline in soil nutrients in the later stages of succession could be attributed to soil degradation. Mt. Jianfengling belongs to a typical tropical rainforest climate, with heavy rainfall, which leads to the loss of soil nutrients with running water (Vitousek and Sanford, 1986), and the soil becomes acidic because of the lack of base ions. This conclusion is also supported by the pH values shown in Table 2. Soil enzymes are one of the most active organic components in soil, and can characterize the capacity of soil metabolism and indicate soil fertility (Garcia-Ruiz et al., 2008). The transformation and cycle of soil nutrients mainly rely on enzyme activity (Hoang et al., 2016). Researchers have found that microbial community composition and enzyme activities are closely correlated (Claassens et al., 2008). The activity of urease and acid phosphatase in this study first increased and then declined along the chronosequence of teak plantations, suggesting that the soil microbial activities changed as plantations grew. Catalase can enzymatically hydrolyze H_2_O_2_, which is beneficial for preventing the toxic effects of H_2_O_2_ on plants (Cavalcanti et al., 2004). The increase in catalase activity observed in this study was similar to that of *Robinia pseudoacacia* plantations reported by Sheng et al. (2017), indicating that the level of oxidative stress in the soil-plant system increased with stand age.

### Change of Arbuscular Mycorrhizal Fungal Colonization and Spore Density With Stand Age

The AM fungal spores were considered to be resting structures that were involved in “long-term” survival (Jing et al., 2020). Spore density in the rhizosphere soil increased linearly along the chronosequence of teak plantations (Figure 1). This result was consistent with the findings of the plantations of *Robinia pseudoacacia* and *Betula alnoides* (Sheng et al., 2017; Jing et al., 2020), which proved that spore abundance in rhizosphere soil was closely associated with the age of the host plant. Pearson correlation analysis showed that spore density was significantly related to soil properties (Supplementary Table 1), suggesting that the increase in spore density with a chronosequence of teak plantation could be mediated by soil parameters induced by stand age. Furthermore, the shift in AM fungal community composition in the rhizosphere soil with age groups may lead to variations in spore density due to the different life histories of different AM fungi (Liu et al., 2009). The colonization rate is an important indicator of the close symbiotic relationship between AM fungi and host plants (Giovannetti and Mosse, 1980). In our study, a linear increase in AM colonization was observed over a chronosequence from 22 to 55 years of age, suggesting that the symbiotic relationship between AM fungi and host plants was enhanced. This result contradicts the report of Jing et al. (2020), who discovered that the change in colonization showed a hump-shaped variation along with a chronosequence, peaking at an intermediate stage of stand age. At present, the change in the colonization rate was not clear over chronosequence. For instance, Zangaro et al. (2013) found that colonization rates decreased along with a chronosequence of ecological succession in southern Brazil, implying that the plants in the early stage of succession were more susceptible to colonization by AM fungi than those in the later stage of succession. These contradictory results may be attributed to the discrepancy in host identity, as physiological states of different host plants vary greatly, and the colonization level is influenced by the host status (physiology and phenology) and edaphic conditions (Liu et al., 2009).

### Changes in Arbuscular Mycorrhizal Fungal Community Diversity and Community Structure

AM fungi are asexual obligate symbiotic fungi with a unique morphology and genome structure, occupying the dual niche of soil and host roots (Vályi et al., 2016). Our results revealed that the richness and diversity of rhizosphere soil and roots did not significantly differ with stand age. Similar to the observations made by Krüger et al. (2017) and Ezeokoli et al. (2020), who reported that the average species richness and diversity (Shannon) in soil and root AM fungal communities had no significant differences over chronological time. This phenomenon may be attributed to the strong association between AM fungi and host plants with succession (Martínez-García et al., 2015). In addition, this phenomenon could also be attributed to the high-throughput sequencing that causes a large variation in sequence abundance obtained between samples. Thus, it is necessary to standardize the samples *via* rarefaction to a common sequencing depth per sample (Hart et al., 2015). Hence, the data obtained from the high-throughput sequencing may not represent the original community (McMurdie and Holmes, 2014). One fact that could not be ignored is that the young stand age (<22 years old) of the teak plantations in our study was missing; therefore, the variation in diversity and richness of the AM fungi community was not observed as succession progressed. A case in point was that the richness (Sobs and ACE) and diversity (Shannon) of the AM fungal community at<1 year were the lowest, and significantly differed from that at all other stages, whereas there was no statistical difference among these stages (5–20 years) (Bi Y.L. et al., 2021). However, our data indicated that richness and diversity were significantly higher in the soil than in the roots (Figure 5), as observed in the study of *Achnatherum inebrians* (Zhong et al., 2021). This result may be explained by the different locations and densities of AM fungal propagules (spores, extra radical mycelium, and infected root fragments) in the soil and roots was different (Varela-Cervero et al., 2016). Furthermore, the host plant could exert a selective force on AM fungi, resulting in preferential symbionts with a subset of the AM fungal taxa that were present in the rhizosphere soil (Bulgarelli et al., 2013). The diversity and richness of root-associated AM fungal communities were significantly positively correlated with P and AP content, respectively (Supplementary Table 3). Pi anions are easily adsorbed by iron and aluminum cations in acidic soils and calcium cations in alkaline soils; therefore, available soil P is generally low on a global scale (Smith and Smith, 2011), especially in tropical forest soils (Sun et al., 2020). P is considered the closest nutrient associated with mycorrhizal formation (Xu X.L. et al., 2020) and a limiting nutrient in terrestrial ecosystems. An increasing number of studies have reported that high levels of P fertilizer application showed a lower richness (Camenzind et al., 2014; Chen et al., 2014) and diversity (Higo et al., 2018) of AM fungal communities because the benefit of the symbiosis was reduced. Conversely, the symbiotic relationship could be enhanced by the limited supply of P (Dueñas et al., 2020). These findings are consistent with the results, suggesting a close relationship between the diversity of the AM fungal community and the P and AP contents.

The NMDS analysis, ANOSIM, and PERMANOVA showed that the composition and structure of the AM fungal community of the teak plantations statistically differed along a chronosequence (Figure 6 and Table 4). A total of 367 and 217 OTUs were detected in soils and roots, respectively (Supplementary Figure 2). They belonged to nine and seven AM fungal genera in soil and root samples, respectively, and AM fungi in the root were a subset of the AM fungi in the rhizosphere soil. These results are in accordance with an earlier report (Sheng et al., 2017). Teak may exert a selective force on the rhizosphere AM fungal taxa, causing differentiation of community composition between the soil and root samples based on the two-step theory (Bulgarelli et al., 2013). Moreover, there is a great difference in the environmental conditions of AM fungi in the roots and soil. AM fungal communities in the roots are mainly determined by the physiological activities of host plants, whereas those in the soil are affected by external environmental factors (Ji et al., 2020). The main AM fungi genera included *Glomus*, *Paraglomus*, *Gigaspora*, *and Rhizophagus* in the rhizosphere soil and root samples (Figure 4 and Supplementary Table 4). *Glomus* was the most dominant in both soil and roots across the chronosequence, which indicated *that Glomus* was an active functional AM fungal taxon. Similar results for the dominance of *Glomus* were observed in teak plantations in Thailand using different methods (Chaiyasen et al., 2014, 2017). The *Glomus* was the most abundant genus reported in forest ecosystems (Chen et al., 2007; Lu et al., 2019; Jing et al., 2020; Ji et al., 2021), reclamation land (Ezeokoli et al., 2020), desert vegetative sites (Vasar et al., 2021), agroecosystems (Wang et al., 2020) and saline ecosystems (Sheng et al., 2019) indicating its wide adaptation to diverse ecosystems. Additionally, studies found that the AMV4.5NF/AMDGR primers favored the amplification of *Glomeraceae* sequences (Luo et al., 2020), which may also have resulted in the dominance of *Glomeraceae* and *Glomus* at the family and genus levels, respectively. Contrary to our results, Sheng et al. (2017) reported that *Rhizophagus* dominated in roots and soils along a chronosequence of blank locust plantations. This discrepancy may be due to different host plant species. For instance, a previous study observed that *Glomus* was most abundant in the rhizosphere soil of mango orchards, whereas *Paraglomus* was most abundant in the rhizosphere soil of litchi orchards (Jiang et al., 2020). *Funneliformis* dominated in the rhizosphere soil of five dominant tree species on the Loess Plateau by a detection method of spore morphology (He et al., 2019). Different detection methods may lead to conflicting results. The method based on spore morphology is not entirely reliable. The subdominant genera in the roots and soils were *Paraglomus* and *Rhizophagus*, respectively. These results were not consistent with several other reports (Herrmann et al., 2016; Ji et al., 2020), which showed that *Acaulospora* and *Diversispora* were the subdominant genera in *Hevea brasiliensis* and mixed broadleaf-conifer forests, respectively.

**TABLE 4:** Analysis of similarities (ANOSIM) and permutation multivariate analysis of variance (PERMANOVA) showing differences in age groups in root-associated and rhizosphere soil AM fungal community compositions between different ages.

Type	Df	ANOSIM		PERMANOVA	
		R	*P*	*F*	*P*
Soil	4	0.74	0.001	1.997	0.001
Root	3	0.37	0.022	1.338	0.025

Species of *Glomus* species have been described as early colonizers (Nielsen et al., 2016). It can improve the ability of host plants to resist pathogens and drought (López-García et al., 2020; Wang et al., 2020). Thus, the increase in the relative abundance of *Glomus* may be related to the enhancement of teak resistance to adverse environments. The decrease in the relative abundance of *Glomus* may be attributed to the improvement in environmental conditions (Figure 5). Moreover, the relative abundance of *Acaulospora*, *Claroideoglomus*, *and Paraglomus* also changed significantly with stand age (*P* < 0.05), and their potential ecological functions deserve further investigation. Moreover, the possible reasons for the shift in composition of AM fungal communities include: (1) compared to those of young plants, the roots of the old plants were longer, thinner, more branched, and had a low nutrient content (Holdaway et al., 2011; Castano et al., 2019), giving more “niches” to AM fungi over the plant ages (Hart et al., 2014). (2) Previous studies have revealed that the plant community can affect the microbial community (Dang et al., 2018); thus, the shift in the vegetation in teak plantations could influence the AM fungal community. Some AM fungal taxa were only detected in specific stand age over chronosequence (Figure 4 and Supplementary Table 4), suggesting that it is feasible for the special AM fungal taxa to act as “indicators” along a chronosequence.

### Factors Driving Arbuscular Mycorrhizal Fungal Communities

Generally, the assembly mechanism of the AM fungal community mainly includes the neutral theory (Hubbell, 2001), ecological niche theory (Dumbrell et al., 2010), and niche-neutral continuum (Gravel et al., 2006). It is generally believed that the community assembly of AM fungi is scale-dependent (Jiang et al., 2018). Soil factors and host plants are the main driving factors for determining the spatial distribution of AM fungi on a local scale (van der Gast et al., 2011). Thus, the community assembly of AM fungal communities in this study may be determined by soil factors, host plants, or both. The Monte Carlo permutation test showed that the K content had a significant impact on the structure of the AM fungal community in soils and roots. Especially in soil, K was the first factor that contributed to 21.7% of the total variation (Figure 8). Several studies have reported that K availability is an important driver of AM fungal community composition due to the accumulation of K content with stand age grew (Qin et al., 2017; Sheng et al., 2017; Luo et al., 2020). High N input can result in the decline of AM fungal abundance, and low N availability conditions can effectively improve the ability of AM fungi to transport N elements to host plants (Xiao et al., 2019). Moreover, AM fungal hyphae could predominantly assimilate N as NH_4_^+^-H, then transported to plants because of the preferential use of NH_4_^+^-H by plants to synthesize amino acids (Govindarajulu et al., 2005). Our study was conducted in an N-limited environment because there were no disturbances (such as fertilization). The relationship between N element transportation between AM fungi and host plants may be enhanced during succession. Consequently, it can be explained that the AM fungal community structure in the rhizosphere soil was significantly correlated with the C/N ratio and NH_4_^+^-H concentration. The Mantel test showed that TOC content had a significant effect on the community structure of AM fungi (Table 3), supporting the above hypothesis.

Evidence in the literature has reported that pH plays a central role in the assembly of soil AM fungal communities (Qin et al., 2015; Wang et al., 2020). The underlying mechanisms may be related to the effect of pH on nutrient availability (Vasar et al., 2021). Due to the major impact of pH on the mobility of multiple substances, the biological processes in the soil were affected (Neina, 2019). Our results showed that pH did not have a significant effect on the structure of the AM fungal community in soil, but had a statistical impact on the structure of the root-associated AM fungal community (Figure 8), suggesting that the structure of the AM fungal community or the processes involved in the AM fungal community in roots may be more sensitive to changes in pH than that in soils. The Mantel and Monte Carlo permutation tests also indicated that the acid phosphatase and catalase activities were closely correlated with the structure of the AM fungal community in soils and roots (Figure 8 and Table 3). Acid phosphatase is involved in soil P cycling, and an earlier study reported the importance of microbial-mediated P cycling (Bi B.Y. et al., 2021). Therefore, the AM fungal communities of the teak plantations were efficient in the decomposition and utilization of inorganic phosphorus by increasing acid phosphatase activity. C/P significantly contributed to the structure of the root-associated AM fungal community by the Mantel test (Table 3), which also partly supported our speculation. This may be closely related to AM fungi obtaining C supply from host plants and compensating the plants by enhancing nutrient acquisition, especially *via* the supply of poorly mobile substances, such as phosphate ions (Dang et al., 2018; Wang et al., 2020). Catalase can decompose H_2_O_2_ and protect host plants and microbes from oxidative damage. Interestingly, it was found that AM fungi could also promote the efflux of H_2_O_2_ from the roots of host plants, thus reducing oxidative stress in host plants under drought stress (Zou et al., 2015). Thus, AM fungi may alleviate oxidative damage in host plants by promoting H_2_O_2_ efflux from host plant roots and increasing catalase activity. The close association between plant communities and the structure of AM fungal communities along succession has been revealed (Krüger et al., 2017). Therefore, further studies are required to investigate the composition of plant communities to systematically and comprehensively analyze how stand age shapes AM fungal communities.

### Implications for Management of Teak Plantations

Tropical rainforests play an important role in the global carbon cycle, especially in the context of global climate change. The tropical rainforest ecosystem is characterized by higher temperatures and much rainfall, suggesting that underground ecological processes and nutrient turnover rates are much faster, such as the decomposition rate of humus and litter mediated by forest soil microbes (Bi B.Y. et al., 2021). Our research reveals that the increase in stand age has no significant impact on the diversity of the AM community in soils and roots. It is not clear from our findings to confirm whether the diversity of AM fungal community changes with succession, since the maximum diversity may have already been reached in the young stage (<22 years) of the teak plantations. However, the number of OTUs detected in soils and roots also peaked at 45Y, showing a trend that first increased and then decreased as stand age progressed (Figure 3). The diversity and complexity of AM fungal communities help maintain the various functions and services of an ecosystem (Li et al., 2021). Hence, regulation of the AM fungal community is essential for the sustainable management of teak plantations.

A significant variation in the composition of the AM fungal community in the soil and roots was observed. The relative abundance of *Glomus* initially increased and then declined, and the relative abundance of *Rhizophagus* increased with stand age (Figure 5). Compared to those of *Glomus*, the species of *Rhizophagus* are more cooperative since they provide host plants with more phosphorus to exchange less carbon, resulting in the host plants preferring to allocate resources to *Rhizophagus* species (Stefani et al., 2020). This phenomenon may be more obvious in the later stages of succession due to a decrease in P availability, increasing the relative abundance of *Rhizophagus*. A typical case is that the transformation of *Glomus* dominated in young stand age to *Rhizophagus* dominated in old stand age (Hart et al., 2014). *Gigaspora* is characterized by the production of extensive hyphae in the soil, promoting the formation of soil aggregation and P uptake (López-García et al., 2020); therefore, it can be understood that the relative abundance of *Gigaspora* increased in the roots due to the decline in P content. The decline in the relative abundance of *Gigaspora* in 55Y is likely to be replaced by other groups with similar functions due to functional redundancy.

Taken together, the number of OTUs detected and structure of the AM community varied with stand age, suggesting that the ecological functions of the AM fungal community are also changing. In a sense of improving the teak plantation’s productivity, fertilization is a direct way to supplement the nutrients needed by the teak plantations in the later stage of succession (45Y), especially phosphate fertilizer. However, the excessive application of phosphate fertilizer may decrease the richness and diversity of AM fungal communities due to the decline in the dependency of host plants for nutrients supplied by AM fungi (Cao et al., 2020). Therefore, the regulation of AM fungal communities by application of biological fertilizers may be feasible in terms of cost-saving and environmental protection.

## Conclusion

The present study demonstrated that soil nutrient content and enzyme activity changed as stand age progressed. It was found that AM fungal spore density and colonization rates increased linearly. Additionally, the increase in stand age significantly changed the structure of the AM fungal communities but had no significant impact on the diversity of the AM fungal communities. In particular, the relative abundance of *Glomus* increased first and then decreased, and the relative abundance of *Rhizophagus* increased with increasing stand age. Our results suggest that age-induced variations in soil properties may be the main force driving the change in the structure of the AM fungal community as teak plantations grew. This study provided a general picture of the diversity and structure of the AM fungal community at different growth stages of teak plantations. Further research is needed to include newly established young stands and screen highly efficient functional AM fungi as biofertilizer inocula to improve the survival rate and productivity of afforested plantations.

## Data Availability Statement

The datasets presented in this study can be found in online repositories. The names of the repository/repositories and accession number(s) can be found below: https://www.ncbi.nlm.nih.gov/, PRJNA743782.

## Author Contributions

KL conceived and designed the study. XW and ML assisted in sample collection. ZY analyzed the data, prepared the figures and tables, and wrote the original manuscript. GH and ZZ helped to improve the manuscript. YC revised and polished the manuscript. All authors contributed to the manuscript and approved the submitted version.

## Conflict of Interest

The authors declare that the research was conducted in the absence of any commercial or financial relationships that could be construed as a potential conflict of interest.

## Publisher’s Note

All claims expressed in this article are solely those of the authors and do not necessarily represent those of their affiliated organizations, or those of the publisher, the editors and the reviewers. Any product that may be evaluated in this article, or claim that may be made by its manufacturer, is not guaranteed or endorsed by the publisher.

## Footnotes

1
http://drive5.com/uparse/

2
http://sourceforge.net/projects/rdp-classifier/

3
http://www.arb-silva.de/

4
http://maarjam.botany.ut.ee/

5
https://www.mothur.org/wiki/Download_mothur
