# Supplementary material for: Alterations in Arbuscular Mycorrhizal Community Along a Chronosequence of Teak (Tectona grandis) Plantations in Tropical Forests of China
Source: Front Microbiol. 2021 Nov 26;12:737068. doi: 10.3389/fmicb.2021.737068 (PMC8660861; doi:10.3389/fmicb.2021.737068)
Supplement: Supplementary file 1 [file Table_1.DOCX]

Supplementary Material

#
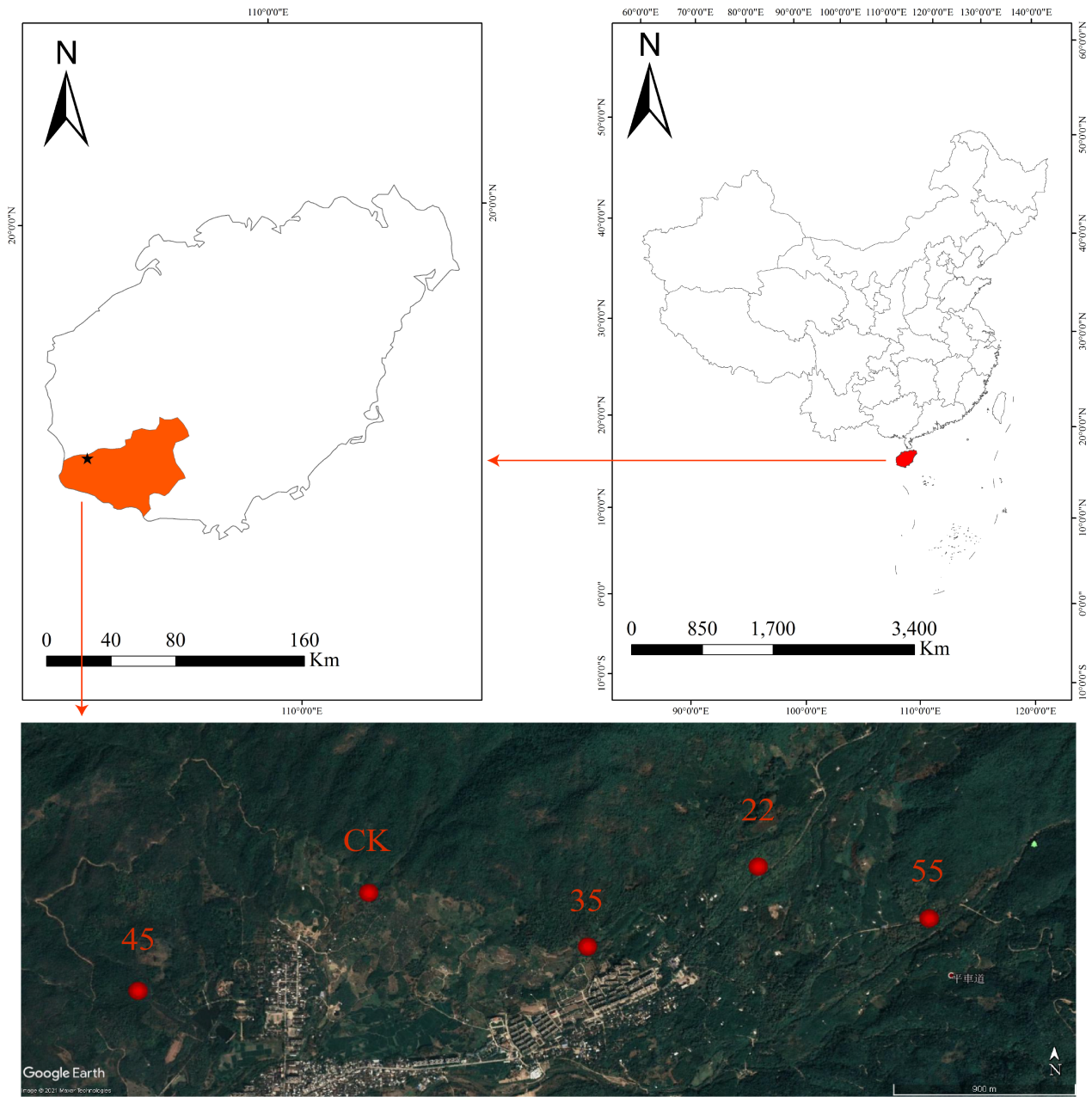


**Figure S1.** Location of sampling plots of teak plantations (22, 35, 45, and 44 years old, and the adjacent grassland – CK) at Mt. Jianfengling in Hainan island, China

**Table S1.** Pearson correlation analysis between soil properties and (1) spore density, and (2) mycorrhizal colonization of arbuscular mycorrhizal fungi.

| Soil property | | Spore density (g^-1^ dry soil) | Mycorrhizal colonization (%) |
| --- | --- | --- | --- |
| pH | 0.66** | | -0.51 |
| SOC | 0.24 | | 0.2 |
| N | 0.59* | | -0.31 |
| P | 0.13 | | 0.13 |
| C/P | 0.18 | | 0.16 |
| C/N | -0.29 | | 0.24 |
| K | 0.092 | | -0.6* |
| AP | -0.26 | | 0.52 |
| AK | 0.62* | | -0.56 |
| NO_3_^--^-H | 0.35 | | -0.59* |
| NH_4_^+^-H | 0.79*** | | -0.85*** |
| Catalase | 0.88*** | | -0.79** |
| Acid phosphatase | 0.38 | | 0.25 |
| Urease | -0.31 | | 0.45 |

SOC: soil organic content; C/P: SOC/P; C/N: SOC/N; N: total nitrogen; P: total phosphatase; AP: available P; K: available potassium; AK: available K; NO_3_^-^-H: nitrate nitrogen; NH_4_^+^-H: ammonium nitrogen. Significant differences by * *p*< 0.05; ** *p*< 0.01; *** *p*< 0.001.

**Table S2.** AM fungal sequences, mean length of sequences, and coverage in each soil and root samples.

| Samples | Soil | | |  | Root | | |
| --- | --- | --- | --- | --- | --- | --- | --- |
|  | Sequence | Mean length | Coverage |  | Sequence | Mean length | Coverage |
| CK-1 | 23038 | 215.97 | 99.96% |  | - | - | - |
| CK-2 | 23114 | 214.64 | 99.96% |  | - | - | - |
| CK-3 | 24126 | 215.37 | 99.97% |  | - | - | - |
| 22-1 | 22891 | 216.62 | 99.96% |  | 20301 | 216.05 | 1 |
| 22-2 | 24386 | 216.13 | 99.96% |  | 17412 | 216.00 | 1 |
| 22-3 | 24035 | 215.84 | 99.97% |  | 23884 | 218.16 | 99.99% |
| 35-1 | 24451 | 215.58 | 99.97% |  | 18638 | 215.83 | 99.95% |
| 35-2 | 22788 | 215.46 | 99.98% |  | 18714 | 215.39 | 1 |
| 35-3 | 21634 | 216.42 | 99.93% |  | 21931 | 215.61 | 99.94% |
| 45-1 | 20492 | 215.61 | 99.96% |  | 19700 | 215.16 | 99.92% |
| 45-2 | 24598 | 216.12 | 99.95% |  | 20091 | 215.28 | 99.94% |
| 45-3 | 24792 | 216.22 | 99.94% |  | 23188 | 215.47 | 99.93% |
| 55-1 | 24074 | 215.77 | 99.99% |  | 22073 | 214.86 | 1 |
| 55-2 | 24403 | 216.37 | 99.93% |  | 23390 | 215.35 | 99.97% |
| 55-3 | 23021 | 216.20 | 99.97% |  | 20743 | 216.25 | 99.98% |


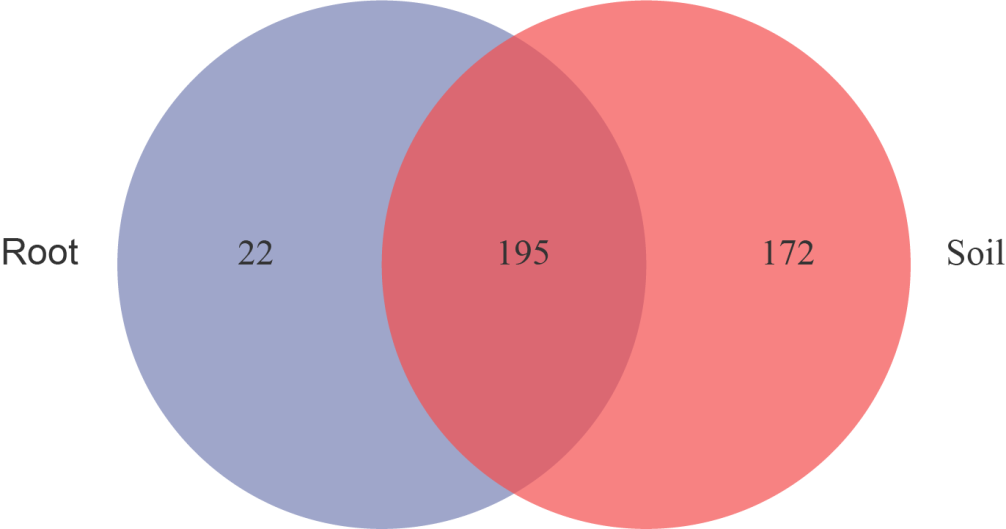


**Figure S2.** The number of AM fungal OTUs unique to and shared between teak roots and rhizosphere soil.


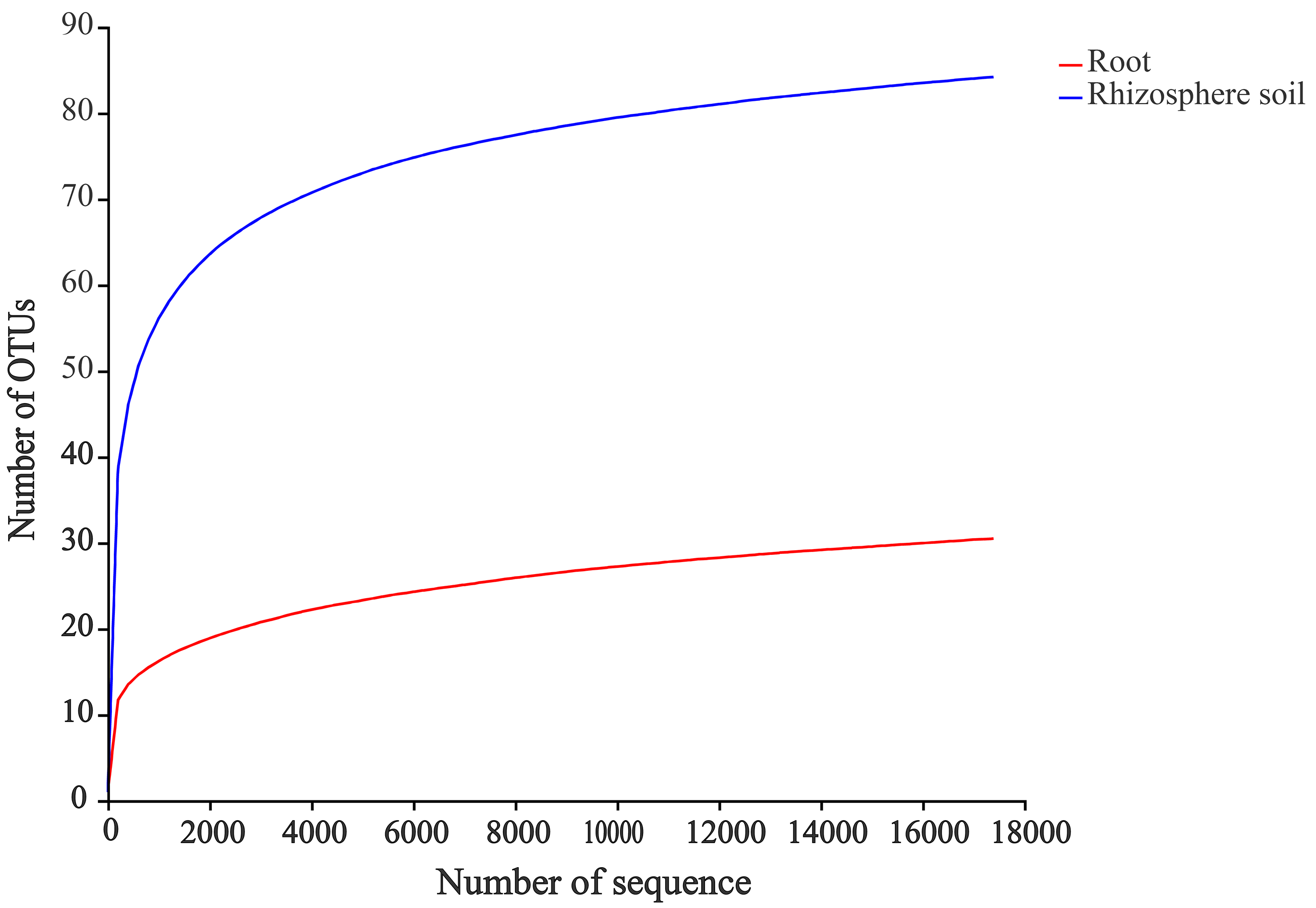


**Figure S3.** Rarefaction curves plotted by teak roots and rhizosphere soils along a teak plantation chronosequence.

**Table S3.** Pearson correlations between soil properties and AM diversity indices in roots and the rhizosphere soil.

| Soil properties | Soils | | |  | Roots | | |
| --- | --- | --- | --- | --- | --- | --- | --- |
|  | Sobs | Shannon | Chao 1 |  | Sobs | Shannon | Chao 1 |
| pH | -0.01 | -0.02 | -0.03 |  | -0.31 | -0.13 | -0.33 |
| SOC | -0.13 | -0.37 | -0.12 |  | 0.44 | 0.43 | 0.45 |
| N | 0.11 | -0.03 | 0.14 |  | -0.05 | -0.07 | -0.04 |
| P | -0.20 | -0.31 | -0.07 |  | 0.57 | 0.75** | 0.53 |
| C/P | 0.02 | -0.18 | -0.09 |  | 0.05 | -0.2 | 0.10 |
| C/N | 0.06 | -0.04 | -0.003 |  | 0.19 | 0.25 | 0.20 |
| K | 0.21 | 0.28 | 0.21 |  | -0.73** | -0.37 | -0.75** |
| AP | -0.37 | -0.48 | -0.20 |  | 0.63* | 0.55 | 0.65* |
| AK | 0.23 | 0.14 | 0.16 |  | -0.51 | -0.21 | -0.53 |
| NO_3_^-^-H | 0.14 | 0.12 | 0.15 |  | -0.27 | -0.021 | -0.3 |
| NH_4_^+^-H | 0.28 | 0.27 | 0.26 |  | -0.52 | -0.25 | -0.56 |
| Catalase | 0.16 | 0.12 | 0.04 |  | -0.68* | -0.27 | -0.72** |
| Soil phosphatase | -0.04 | -0.26 | 0.06 |  | 0.37 | 0.42 | 0.38 |
| Urease | -0.39 | -0.48 | -0.20 |  | 0.70* | 0.55 | 0.71* |

SOC: soil organic carbon; N: total nitrogen; P: total phosphorous; AP: available P; K: potassium; AK: available K; C/P=SOC/P, C/N=SOC/N; NH_4_^+^-H: ammonium nitrogen; NO_3_^-^-H: nitrate nitrogen. Significant differences by * *p*<0.05; ** *p*<0.01.

**Table S4.** Relative abundance of AM fungi in the teak rhizosphere soils and roots along the chronosequence of teak plantations. Values are mean (n=3).

| Stand age (year) | | CK | 22Y | 35Y | 45Y | 55Y |
| --- | --- | --- | --- | --- | --- | --- |
| Soils | *Glomus* | 56.89 | 74.81 | 77.02 | 76.63 | 55.60 |
|  | *Rhizophagus* | 5.13 | 2.64 | 6.02 | 5.30 | 12.12 |
|  | *Gigaspora* | 16.81 | 0.05 | 1.06 | 2.41 | 4.85 |
|  | *Acaulospora* | 0 | 0.01 | 0 | 1.29 | 9.24 |
|  | *Septoglomus* | 1.09 | 4.23 | 5.01 | 5.26 | 4.21 |
|  | *Paraglomus* | 6.63 | 15.46 | 9.58 | 7.33 | 7.47 |
|  | *Scutellospora* | 9.10 | 1.15 | 0 | 0.08 | 0.01 |
|  | *Claroideoglomus* | 4.13 | 1.49 | 1.31 | 1.70 | 5.52 |
|  | *Diversispora* | 0.22 | 0.18 | 0 | 0 | 0.98 |
| Roots | *Glomus* | - | 69.23 | 77.03 | 69.21 | 60.10 |
|  | *Rhizophagus* | - | 15.70 | 14.71 | 13.59 | 25.10 |
|  | *Paraglomus* | - | 13.99 | 1.67 | 0.04 | 1.88 |
|  | *Gigaspora* | - | 0 | 5.65 | 13.00 | 5.10 |
|  | *Scutellospora* | - | 1.07 | 0.15 | 1.62 | 3.12 |
|  | *Septoglomus* | - | 0 | 0.79 | 2.05 | 0.92 |
|  | *Claroideoglomus* |  | 0 | 0 | 0.73 | 3.77 |

22Y: 22 years old stand; 35Y: 35 years old stand; 45Y: 45 years old; 55Y: 55 years old stand; CK: the adjacent grassland.
